# Design, monitoring and evaluation of a direct payments approach for an ecotourism strategy to reduce illegal hunting and trade of wildlife in Lao PDR

**DOI:** 10.1371/journal.pone.0186133

**Published:** 2018-02-28

**Authors:** Paul Frederick Eshoo, Arlyne Johnson, Sivilay Duangdala, Troy Hansel

**Affiliations:** 1 Wildlife Conservation Society–Lao PDR, Vientiane Lao PDR; 2 Foundations of Success, Bethesda MD United States of America; U.S. Geological Survey, UNITED STATES

## Abstract

Ecotourism as a strategy for achieving biodiversity conservation often results in limited conservation impact relative to its investment and revenue return. In cases where an ecotourism strategy has been used, projects are frequently criticized for not providing sufficient evidence on how the strategy has reduced threats or improved the status of the biodiversity it purports to protect. In Lao PDR, revenue from ecotourism has not been directly linked to or dependent on improvements in biodiversity and there is no evidence that ecotourism enterprises have contributed to conservation. In other developing countries, direct payments through explicit contracts in return for ecosystem services have been proposed as a more cost-effective means for achieving conservation, although further research is needed to evaluate the impact of this approach. To address this need, a new model was tested in the Nam Et-Phou Louey National Protected Area (NPA) in Lao PDR using a direct payments approach to create ecotourism incentives for villagers to increase wildlife populations. Over a four-year period, we monitored along a theory of change to evaluate assumptions about the linkages between intermediate results and biological outcomes. Preliminary results show a negative correlation between ecotourism benefits and hunting infractions in target villages; no increase in hunting sign in the ecotourism sector of the NPA relative to a three-fold increase in hunting sign across the NPA’s non-tourism sectors; and an overall increase in wildlife sightings. This case provides key lessons on the design of a direct payments approach for an ecotourism strategy, including how to combine threat monitoring and data on wildlife sightings to evaluate strategy effectiveness, on setting rates for wildlife sightings and village fees, and the utility of the approach for protecting very rare species.

## Introduction

The effectiveness of ecotourism as a strategy for achieving biodiversity conservation using an indirect payments approach, which is typical of alternative livelihood projects, has been identified as largely unsuccessful in demonstrating measurable conservation outcomes [1, 2]. The assumption of an indirect payments approach is that increased income from an ecotourism operation will result in those benefitting from ecotourism (e.g., communities, private sector, etc.) placing a greater value on biodiversity and acting to conserve it—without an explicit agreement to do so [3, 4]. Implicit in this assumption when applied in developing countries is that rising incomes from ecotourism will contribute to poverty reduction that will lead to reduced dependence on natural resources as well as increased support from ecotourism beneficiaries for regulations to conserve the biodiversity upon which the ecotourism is based. It also implies that beneficiaries will accurately interpret and act upon the wishes of tourists that are paying for the ecotourism activity in part to support biodiversity conservation. There is valid concern that ecotourism projects have typically not provided evidence to validate the assumption that ecotourism reduces threats and leads to positive changes in the status of biodiversity, which leaves conservation project managers with no clear guidance as to whether or not an investment in ecotourism as a strategy for achieving conservation is effective or not [5, 6].

In Lao PDR (hereafter Laos), where illegal hunting and trade is driving wildlife decline [7, 8] an indirect payments approach has been used by many ecotourism projects hoping to reduce this threat by alleviating poverty in villages surrounding national protected areas (NPAs) [9]. Thus far only one study has evaluated the impact of these ecotourism projects on wildlife conservation [10]. The study found that the abundance of western black-cheeked crested gibbon (*Nomascus concolor)*, the target species that the ecotourism project aimed to conserve, continued to decline due to illegal hunting and trade despite an increase in ecotourism income to communities in the ecotourism area. Surprisingly few studies have examined the impact of ecotourism in protected areas on illegal hunting in tropical forests. Of those that have, most report that tourism revenues were used to reduce poaching pressure by supporting socioeconomic benefits in the form of employment, education or community development for local groups involved in the illegal hunting [11–16]. In most cases, the studies reported that poaching had continued and recommended an arrangement that would better link benefit-sharing from tourism to compliance with hunting regulations to reduce illegal hunting [12, 17, 18]. In other developing countries, direct payments through explicit contracts in return for ecosystem services have been proposed as a more cost-effective means for achieving conservation [19, 20], although they caution that further research is needed to evaluate the impact of direct payments on achieving conservation results [21–23].

To address this need, in this paper results are presented from an ecotourism strategy designed to directly link the number and type of wildlife sighted by tourists with the amount of financial benefits received by beneficiaries involved in an ecotourism operation with the ultimate goal of increasing wildlife abundance in the ecotourism area. This strategy builds on a similar ecotourism model of direct payments piloted in nearby Cambodia [24], but is unique in that it shares benefits with multiple villages, giving incentives to all families that have access to the ecotourism area where hunting is prohibited and targets a variety of wildlife species by using a tiered pricing system, with the purpose of protecting carnivores, ungulates and primates that are declining due to illegal hunting and trade. Benefits were designed to increase incrementally according to the number of animals sighted by visitors in order to provide greater return for increases in wildlife abundance. Results from the first four years of the ecotourism operation (2009–2013) are presented, along with a simple and replicable monitoring system that was used for evaluating the effectiveness of the ecotourism strategy. Our findings have implications for the design of other payment for ecosystem services (PES) ecotourism models and are important for conservation practitioners that are considering investments in ecotourism as a strategy to reduce illegal hunting.

### Study area

**Fig 1:**
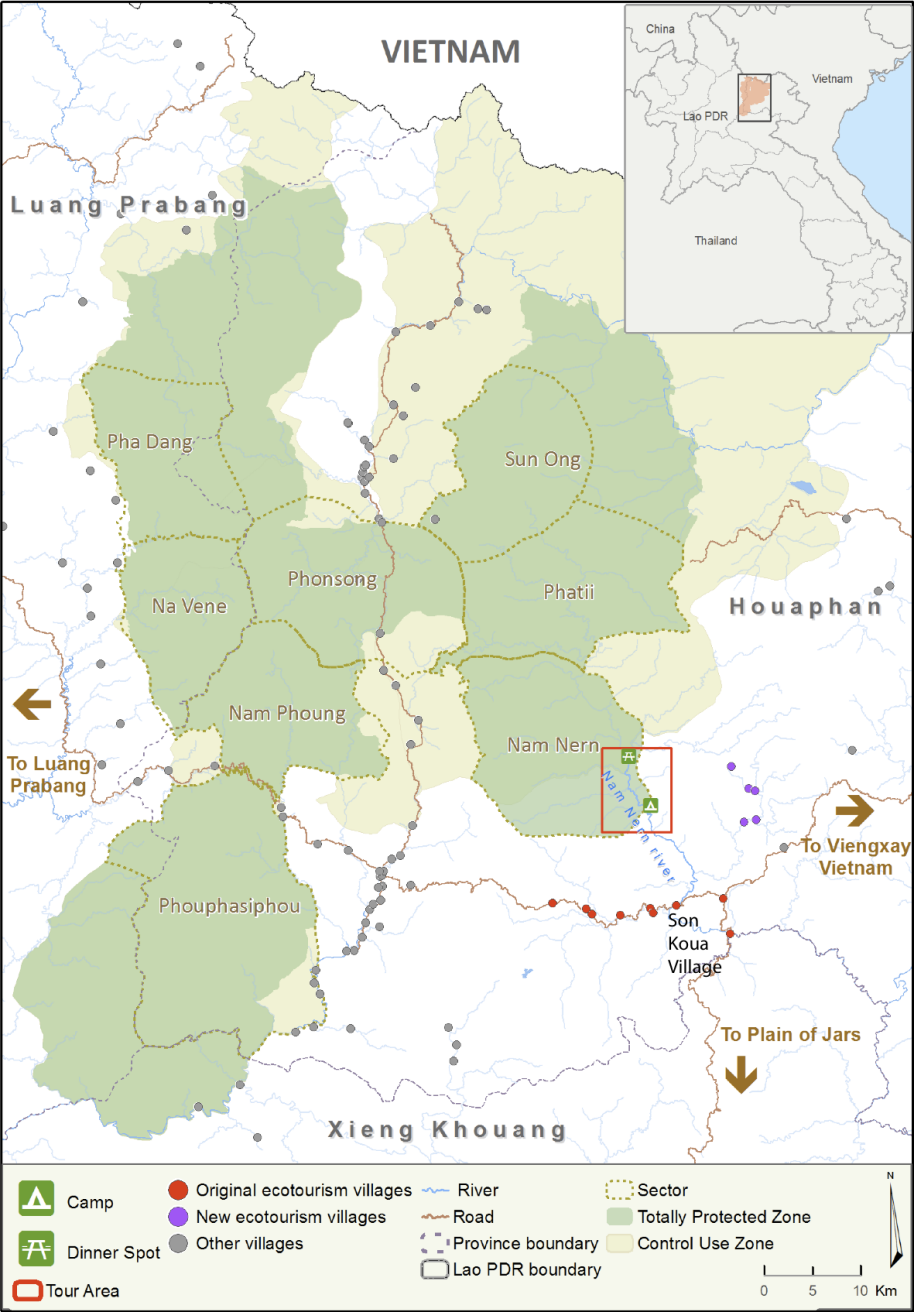
Map of the 4,229 km2 Nam Et-Phou Louey National Protected Area, Laos.

The 4,229 km^2^ Nam Et-Phou Louey (NEPL) NPA is located in the northern highlands of Laos [Fig 1]. At a global level, NEPL is an important representative of the Northern Indochina Subtropical Forests Ecoregion [25] and one of the largest protected areas in the ecoregion with high biological diversity and many charismatic species. Ecotourism was introduced as a strategy to reduce illegal hunting and trade, which was the principle threat contributing to wildlife decline in the NPA [26, 27]. This threat was driven by international demand for tiger bones, bears, pangolins, and primates with additional demand from urban markets in Laos for wild meat (e.g., ungulates and large rodents)[28]. Evidence gathered from camera trap surveys, focal group discussions and law enforcement patrols indicated that the hunters were primarily from villages bordering the NPA with access to illegal weapons—including guns, explosives, and traps [26, 29–31]. There was little evidence of hunters coming from beyond the NPA, which was likely due to the remote and rugged nature of the heavily forested and mountainous landscape. Elevation ranges from 400-2257m with 91% of the area along slopes greater than 12%. It was even uncommon for patrol teams to encounter hunters outside of their respective village sector. Of the 84 hunter groups caught by patrol teams in 2009, only five were from another village sector and one from a non-NPA village. Villagers hunting for large mammals deep in the forest were typically in groups, while people tending to upland rice fields and grazing livestock in satellite locations inside the forest hunted alone or in small pairs [31, 32]. Buyers were normally influential villagers that acted as local middlemen, selling their products to other Lao traders from outside the province, or foreign traders from Vietnam or China [27]. Houaphan Province, where the ecotourism site was located, was one of the poorest provinces in the country [33], with 41% of its population in poverty [34]. The average annual household income for villages around the NEPL NPA was USD436-618 (at 8,000 Lao Kip/USD) and the total expenditure per capita by the government and international development projects on public services in the province was USD38 [35].

The ecotourism site was located on the Nam Nern River in the NEPL NPA (Fig 1), which was identified as a feasible location for developing wildlife-based tourism because it provided a unique opportunity to see wildlife [36], which was relatively uncommon elsewhere in Laos [7]. This was due in large part to an NPA law enforcement strategy implemented in 2005 [31], and the river that allowed for stealthy boat travel to view wildlife visiting the river for water and minerals. The location was also identified as a viable tourism development area as it is situated at the crossroads of three major tourist destinations: the UNESCO World Heritage site of Luang Prabang Town, the UNESCO World Heritage-nominated Plain of Jars in Xieng Khouang Province, and the Pathet Lao Caves in Viengxay, Houaphanh Province and road to Hanoi [36].

## Materials and methods

### Theory of change

The overall goal of NEPL NPA was to increase the abundance of globally important wildlife populations in the NPA [27]. In designing the ecotourism strategy, it was assumed that the NPA law enforcement strategy, which includes fines on individual hunters and wildlife traders for breaking NPA regulations, was not adequate to completely remove the threat of illegal hunting and trade—an assumption supported by law enforcement monitoring results [31]. It was believed that by adding additional individual and communal economic incentives through ecotourism that these threats could be further reduced and wildlife populations increased. It was assumed, however—based on evidence from Laos [10] and elsewhere [1, 37, 38]—that simply introducing ecotourism and generating income for local people in one or more villages nearby the tour area would not necessarily result in greater protection of wildlife for several reasons. Among these reasons were that: (i) not all tourists are attuned to wildlife conservation issues and may elect to go on tours and generate income for villagers irrespective of whether or not wildlife populations increase or poaching decreases; (ii) villagers working in ecotourism may continue to hunt when there are no tourists in the area because they like hunting and/or will hunt and sell wildlife to earn more money over and above their ecotourism income; and iii) hunters from villages not receiving ecotourism benefits may continue hunting in the tour area.

**Fig 2:**
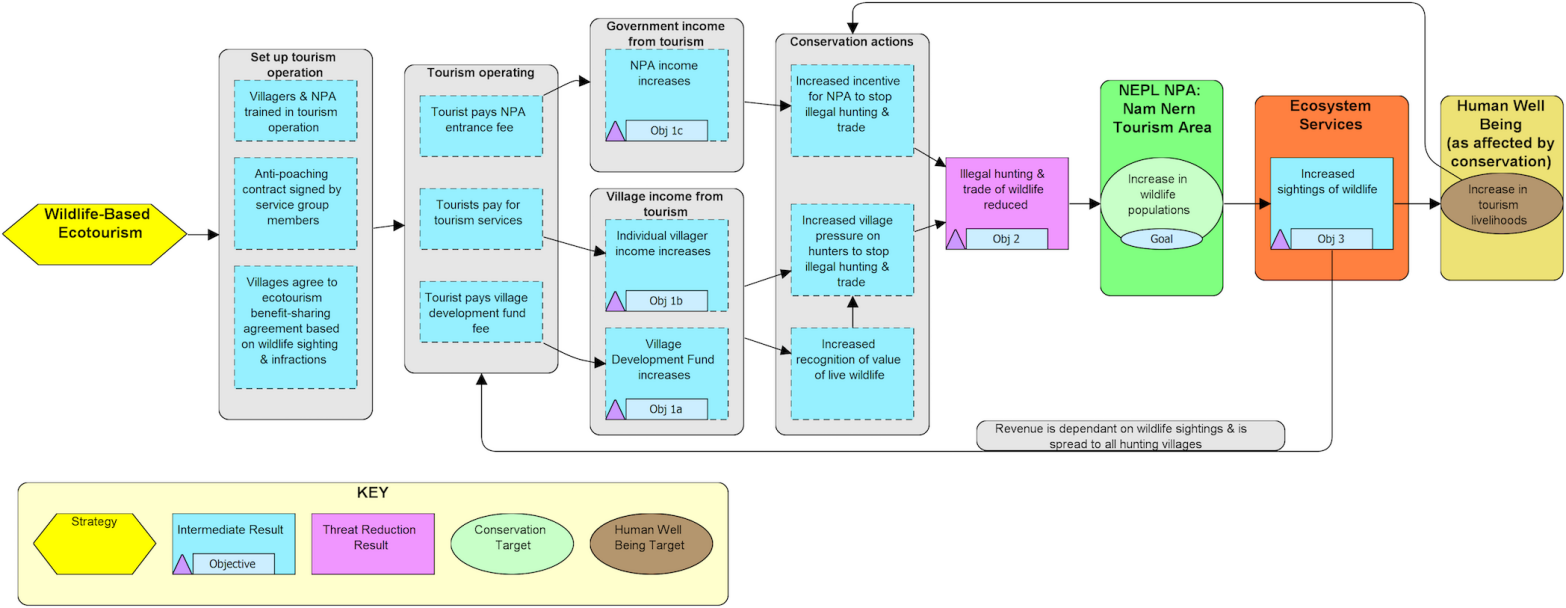
Results chain illustrating the theory of change for a direct payments model for wildlife-based ecotourism strategy. The assumptions of the model are that increased income from ecotourism will lead to a reduction in illegal hunting and trade that will contribute to increased sightings of wildlife and an increase in tourism livelihoods, thus creating a positive, synergistic loop for wildlife protection. (Created in Miradi 4.4.0).

Given this reasoning, our theory of change (TOC), which is a string of expected outcomes that result from implementing a conservation strategy [39, 40] [Fig 2] hypothesized that if all potential hunters have an economic stake in protecting wildlife by sharing the financial benefits of ecotourism, and if these benefits are pegged to the actual numbers of wildlife viewed by tourists, then a positive loop of increasing benefits and wildlife could be created. It was hypothesized that if villagers could see that the ecosystem services resulting from wildlife conservation, including increasing wildlife populations and the consequent increase in tourist sightings of wildlife that resulted in more income, then a desire for more benefits would cause them to reduce illegal hunting in the tour area. It was predicted that over time with tourists visiting and paying to see wildlife, wildlife sightings on tours would increase and that illegal hunting and disturbance in the NPA’s Totally Protected Zone (TPZ) would decline as financial benefits generated by ecotourism increased.

### Developing the ecotourism model

A business plan was completed in 2009 to determine the financial feasibility of an ecotourism operation in the NPA [36]. Based on the plan, the NPA, district government and the Wildlife Conservation Society (WCS) chose to develop one ecotourism product, the “Nam Nern Night Safari”, to test the assumptions of the model. The product involved riding boats upriver inside the NPA’s TPZ during the day and floating downriver at night to view wildlife by spotlight with the assistance of village guides [Fig 1]. The tour was set up with a six-person, three-boat maximum (two people per boat), which was later increased to ten persons and four boats (three people per boat). On tours with more than one boat, departure times for the night-time, wildlife-spotlighting float downriver were staggered by approximately thirty minutes to one hour in order to provide tourists with a roughly equal chance of seeing wildlife. A set itinerary was followed, starting and ending at the same locations at approximately the same times for each tour. Such conditions also allowed for comparison of data collected on wildlife sightings from each boat.

#### Contract design

A species-specific contract [21] was developed with the local government and villages in the ecotourism area, which stated how benefits from ecotourism would be distributed and the conditions under which ecotourism would be managed. Benefits included a shared fund that established an explicit positive relationship between the fund and numbers of wildlife seen by tourists and a negative relationship between the fund and the number of infractions of NPA regulations committed by villagers. A mechanism was also developed that discouraged villagers working in tourism to illegally hunt or trade wildlife. All villages with legal access to the tour area under the State’s land allocation process[41] were identified, which included nine original villages from the Tai-Kadai and Mon-Khmer ethno-linguistic groups [Fig 1], a total of 5,071 people (859 households; range of 45–156 households per village). Most households engaged in subsistence agriculture and had limited opportunities for earning cash income and participating in the market economy [27]. The project held ecotourism and conservation seminars in each village to raise awareness among villagers and all potential hunters about the need for protection of the natural attraction—wildlife and their habitat—in order to make the ecotourism operation a future success that would generate reoccurring income for their villages. The contract, which stated how benefits from ecotourism would be distributed and the conditions under which ecotourism would be managed, was negotiated with the nine villages, the district government, and the NPA. As every family in each village was assumed to have equal access to the TPZ, it was required that all families be consulted and agree to the benefit-sharing agreement. Finally, the contract was signed by all village chiefs, the district governor, and the head of the NPA to make it legally binding.

#### Definition of biodiversity conservation services and benefit distribution

The benefit distribution contract was used to clearly define the expected conservation services and how benefits would be distributed [21]. This specified how ecotourism income would be dispersed among the nine villages through a village development fund (VDF) and how tourism services would be provided through village tourism service groups (guides, cooks, boat operators, lodge managers and handicraft producers) in the one village (Son Koua) positioned at the start of the river trip [Fig 1]. Separate contracts were also signed with each individual of the tourism service groups, which stated that they would lose their position in the service group if they or anyone in their family (as registered in their official family registration book) were caught violating NPA regulations. With the VDF, the villages were guaranteed a specified amount of money for every individual tourist going on the tour. In addition to this, for every listed species of wildlife seen by a group of tourists, an additional bonus would be paid into the VDF in order to create an explicit incentive for conservation [Table 1].

**Table 1:** Bonuses paid into village development fund for each individual tourist on the tour and for the three different classes of wildlife seen per boat while on the tour.

Class	Common Name; Species	IUCN Status*	Bonus–Total (USD)			Bonus per Village (USD)		
			Y1	Y2	Y3-4	Y1	Y2	Y3-4
Class I	Tiger *Panthera tigris*	EN	225.00	225.00	262.50	25.00	25.00	18.75
Class II	Tiger tracks; *Panthera tigris*	EN	2.25	5.00	4.38	0.25	0.50	0.31
	Asian Golden Cat; *Pardofelis temminckii*	NT						
	Otters; *Aonyx cinerea*, *Lutrogale perspicillata*, *Lutra lutra*	VU, NT						
	Sambar; *Cervus unicolor*	VU						
Class III	Lorises; *Nycticebus spp*.	VU	1.13	2.25	2.19	0.13	0.25	0.16
	Macaques; *Macaca spp*.	VU, NT						
	Leopard tracks; *Panthera pardus*	NT						
	Muntjacs; *Muntiacus sp*.	LC, DD						
	Civets; *Hemigalus owstoni*, *Viverra zibetha*, *Viverricula indica*, *Paguma larvata*, *Paradoxurus hermaphroditus*, *Arctogalidia trivirgata*	VU, NT, LC						
	Hog Badger; *Arctonyx collaris*	NT						
	Southwest China Serow; *Capricornissmilneedwardsi*	NT						
	Porcupines; *Hystrix brachyura*, *Atherurus macrourus*	LC						
	Monitor Lizards; *Varanus bengalensis*, *V*. *salvator*	LC						
Tourists	* *		5.63	11.25	10.94	0.63	1.25	0.78

* Globally threatened-endangered (EN); Globally threatened-vulnerable (VU); Globally near threatened (NT); Data deficient (DD); Least concern (LC) [18]

Bonuses for wildlife sightings were split into three classes, each with a unique rate based on their relative rarity in the NPA [Table 1] to create greater incentives to protect rarer and more threatened species. Class I bonuses were for tiger sightings, the NPA’s flagship species [27]. Class II bonuses included sightings of tiger tracks and the NPA’s other target species (Asian golden cat, otters, and Sambar deer). Class III included other threatened or relatively uncommon species that were observed during baseline surveys.

Nominal values for Class II and Class III bonuses and for tourist entry fees were determined by first setting the maximum amount to be paid by tourists for the cumulative sum of all such bonuses (not including a tiger sighting). This maximum amount was called the VDF fee and was paid up front by tourists as part of the all-inclusive tour price. It was reasoned that having tourists pay up front for bonuses in an all-inclusive fee would reduce both the complexity for tourists to pay for the tour by setting a fixed tour price and also reduce the risk of tourists under-reporting wildlife sightings. The VDF fee was based on market research of retail prices for similar tour products in the region by Bhula et al. [36], who suggested a rate of USD10 per person. The rate for each individual tourist was set at half of the VDF fee, reasoning that it would be fair to guarantee that at least 50% of VDF earnings would be paid to the villages in the event that there were no wildlife sightings on a tour. The amount to be paid for Class II and Class III bonuses was calculated from the average number of sightings per boat trip during baseline surveys prior to the start of the tourism operation, which resulted in a 1:2 ratio of the Class II bonus rate to that of the Class III bonus rate—a proportion that was deemed reasonable and easy for villagers to understand.

The bonus for a tiger sighting was set much higher than the others in an attempt to create an extraordinary incentive for such observations, which had been made by project staff only a few times since 2003. The rate was based on the project’s perception of what may seem like a sizeable payment to villagers (approximately half of a household’s average annual income [21]) and what could reasonably be paid by a group of tourists or by the project itself if tourists were not willing to pay this fee above the standard cost of the tour. (Later in the project, it was determined that the bonus for tiger sightings should be included in the fixed VDF fee, i.e. tourists would not have to pay extra for a tiger sighting because the probability of a tiger sighting was so infrequent that it could be covered by the standard VDF earnings.)

In addition to including these positive incentives for conservation in the ecotourism strategy design, disincentives for breaking NPA regulations were also created by the benefit distribution contract. If anyone from an ecotourism village were caught by NPA law enforcement teams violating regulations, the VDF of the respective villages of these individuals would be reduced for the year. For the first infraction the annual VDF would be reduced by 25%, by 50% for the second, and by 100% for three or more infractions. Penalties were assessed per annum, and a village was allowed to begin accumulating ecotourism benefits again in the next tourism season. Each village chose by popular vote one development activity that could benefit the entire community for which to use their funds at the end of the tourist season (June–August). Examples of activities chosen included medicine for a revolving medicine bank, materials to fix or build a school structure, and new benches for community meeting halls. In Year 3, most villages voted to use the VDF for their existing revolving microfinance fund instead of purchasing materials for the communal project.

### Evaluating the effectiveness of the ecotourism model as a strategy to reduce illegal hunting and trade

The goal of the ecotourism strategy was to increase threatened wildlife populations in the NEPL NPA, by increasing wildlife in the Nam Nern sector of the NPA’s TPZ where the ecotourism operation was located [Fig 1]. To achieve this goal, the objectives of the strategy were to initially i) increase village and NPA income from ecotourism; and then ii) reduce illegal hunting and trade of wildlife in the Nam Nern sector; and ultimately iii) increase sightings of wildlife by tourists on the Nam Nern Night Safari [Fig 2]. To assess the effectiveness of the ecotourism model, the project annually measured and evaluated progress towards these three main objectives. This aligned with the standard recommendations for performance monitoring of conservation agreements, which includes regular and systematic assessment of socio-economic impact, compliance with agreements, and status of biodiversity targets [21].

#### Ecotourism income

The village and NPA incomes from ecotourism (Fig 2, Objectives 1a-c) were monitored by NPA staff that booked and led tours. Data were compiled on all tour revenues and expenses and were summarized according to three income types including i) VDF income that was distributed equally among all villages; ii) village service group income, which was shared by 23–41 individual families that work in tourism from Son Koua Village; and iii) NPA entrance permit fees, which were used by the NPA to support the implementation of various conservation strategies (e.g., law enforcement, conservation outreach, ecotourism, etc.).

#### Compliance with agreements

Illegal hunting and trade of wildlife (Fig 2, Objective 2) was systematically monitored by NPA patrol teams in eight sectors of the TPZ (Fig 1) using a MIST law enforcement monitoring system [31, 42]. Each sector was approximately 266 km^2^ in size and was covered regularly by an eight-member patrol team. During daily foot patrols, the team employed a standardized protocol to collect data on hunting signs, including hunters, weapons and gear, hunting camps encountered and gunshots heard. The impact of the ecotourism strategy on reducing illegal hunting was evaluated by comparing change in hunting catch per unit effort (CPU; hunting signs relative to patrol effort measured in kilometres walked), from the baseline year to each subsequent year following the introduction of the ecotourism operation in the Nam Nern sector of the TPZ, against change in hunting CPU in five other TPZ sectors (Pha Dang, Na Vene, Phonsong, Nam Phoung, and Phouphasiphou, see Fig 1) where ecotourism was not implemented. Infractions, defined as incidents recorded by NPA enforcement staff when individuals or groups were arrested for breaking NPA regulations, were used to calculate reductions in ecotourism benefits for the VDF and service providers, and as an additional indicator of the level of threat at the ecotourism area.

#### Status of biodiversity

Wildlife sightings by tourists (Fig 2, Objective 3) were recorded at the end of each tour. Tourists used a standardized wildlife monitoring form provided by the NPA to record, as a group, the numbers of wildlife seen during the tour. Tourists were instructed to record only wildlife they actually saw and not wildlife seen only by guides. The total number of sightings per group was divided by the number of boats per group to calculate the average sightings per boat. Wildlife sightings by tourists were compared with baseline data collected by project staff in the tour area in the year preceding the opening of the ecotourism operation. All monitoring data were compiled and reported annually for the period of June 1-May 31.

## Results

### Ecotourism income

**Fig 3:**
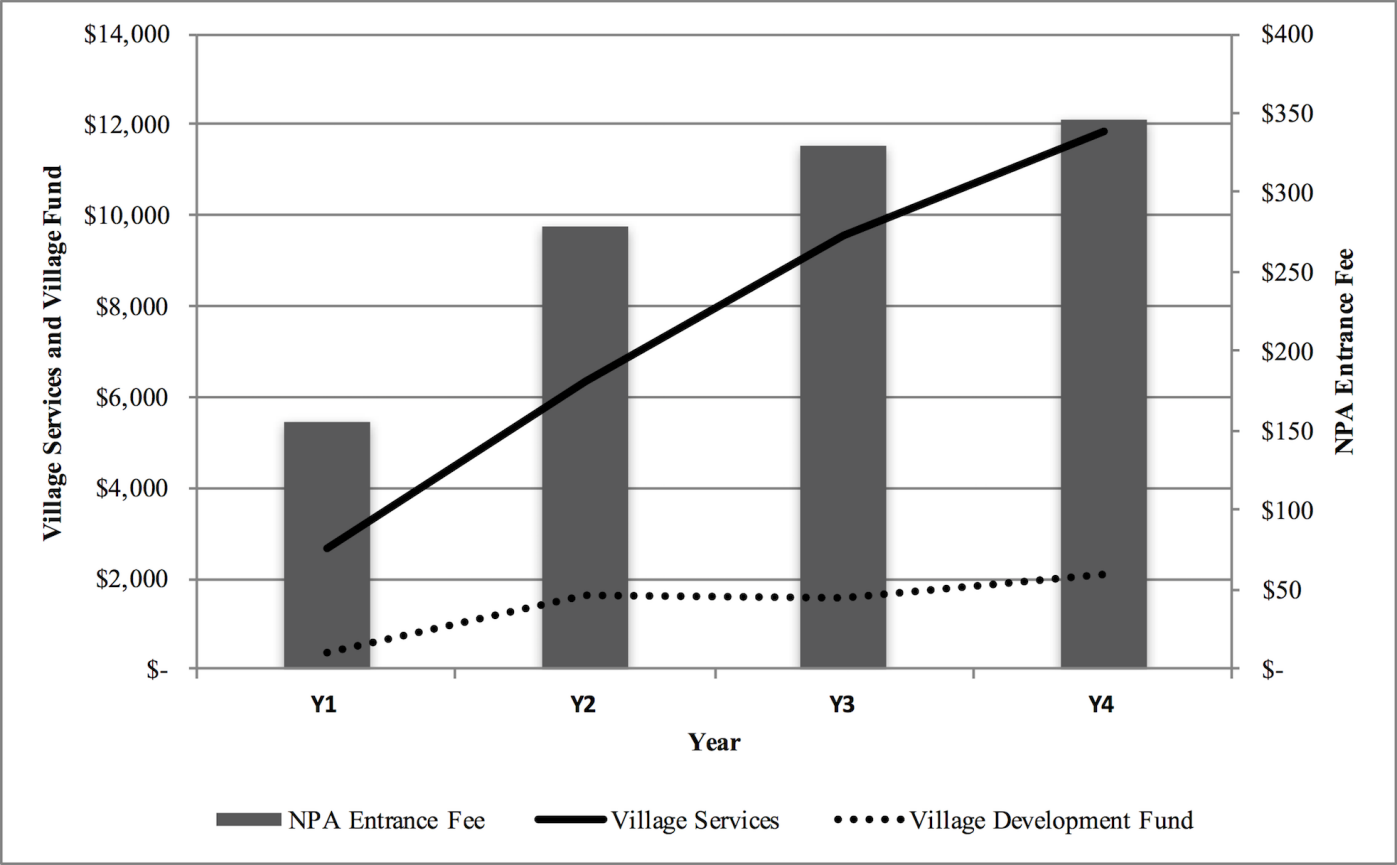
Income from ecotourism during the first four years of operation of the Nam Nern Night Safari (2010–2013) for village services and village funds (left axis) and NPA entrance fees (right axis).

**Fig 4:**
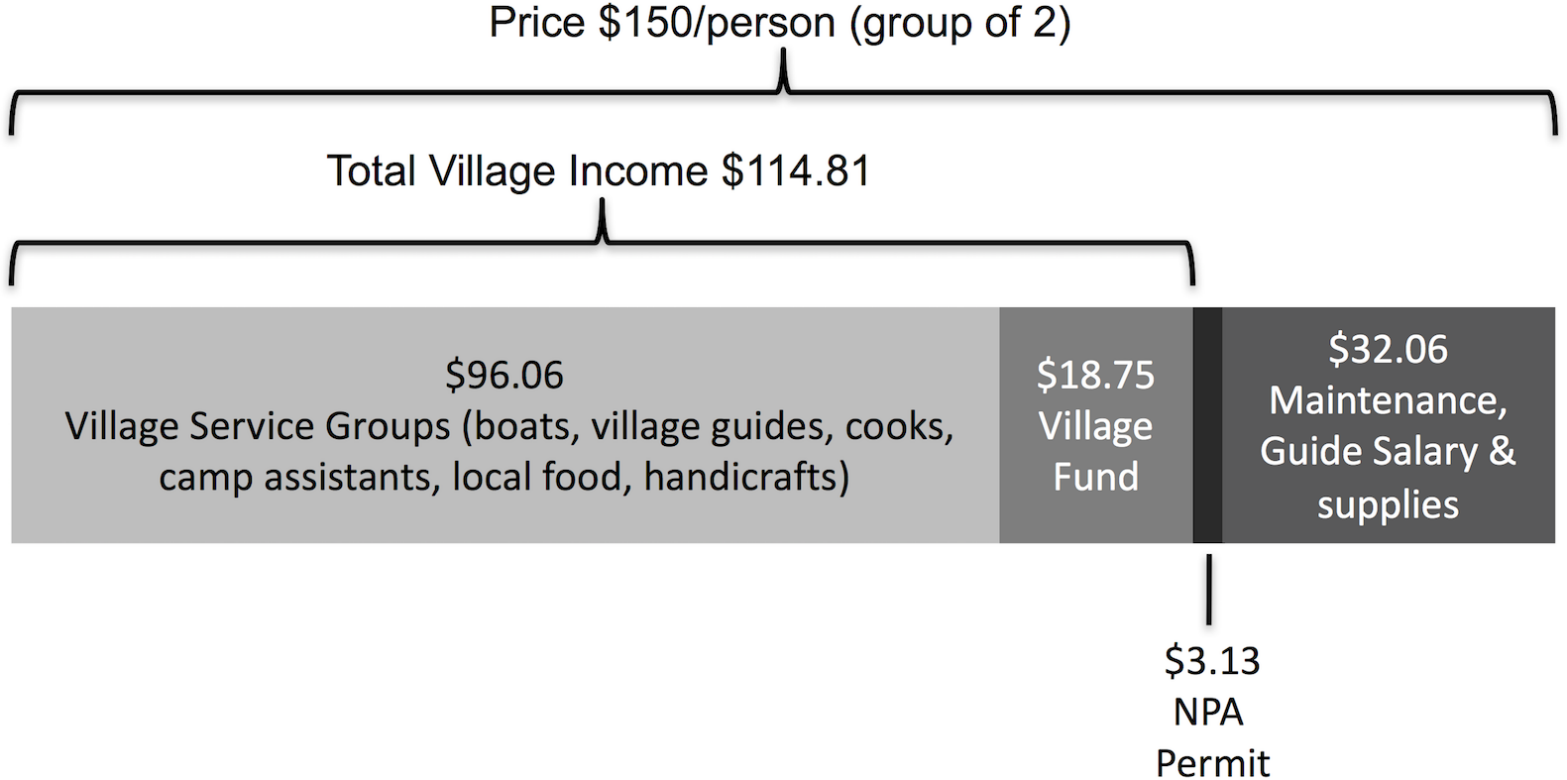
Revenue distribution from the Nam Nern Night Safari (approximate calculation for a group of 2 tourists using costs and prices from years 2 and 3).

During the first four years of the ecotourism operation (2010–2013), a total of 367 visitors took the Nam Nern Night Safari. Relative to the 2010 baseline, there was a two-fold increase in visitors per annum (2.2; range 50–116), a four-fold (4.4) increase in earnings of the village tourism service groups, and a five-fold (5.5) increase in the VDF [Fig 3]. Each tour group of two people at a price of USD150 per person generated USD114.81 for villages and USD3.13 for the NPA [Fig 4].

### Compliance with agreements

**Fig 5:**
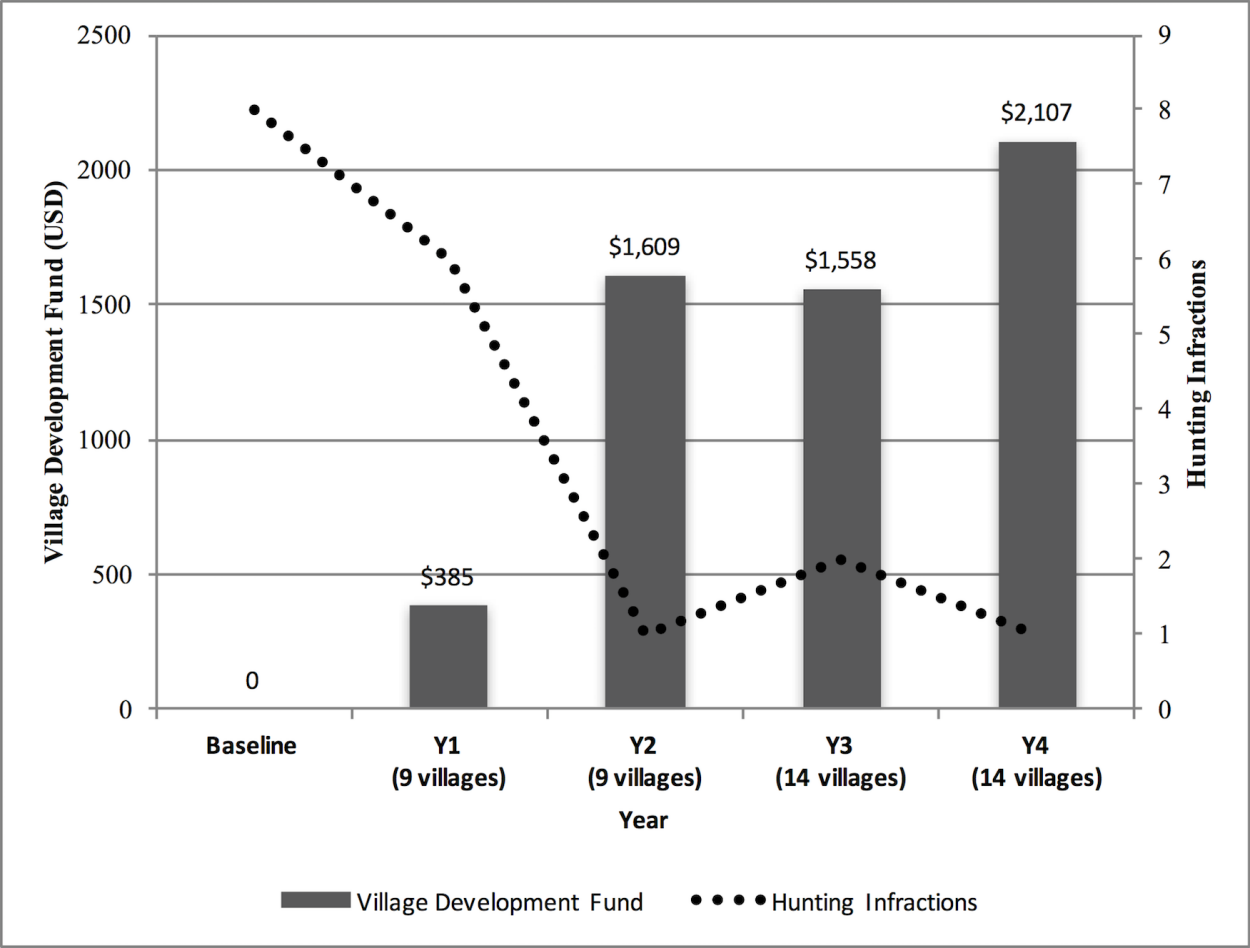
Relationship between village development fund income and hunting infractions by villages in the Nam Nern ecotourism area (the Nam Nern sector of the protected area) from 2009–2013.

Across the five-year period (baseline plus the four years with tourism), there was a negative correlation between the annual VDF and hunting infractions in the tourism villages (r = -0.98, n = 5, p = 0.01) [Fig 5]. In Year 1, five villages, including Son Koua. had no infractions and qualified to receive the full amount of their VDF. Two villages had one infraction each and received 75% of their VDF while the remaining two villages had two infractions each and received only half of their VDF.

In Year 2, eight of nine villages had no infractions. The only hunting infraction was committed by villagers from Son Koua, which was the illegal harvest of a large wild felid (a leopard or tiger; evidence was inconclusive), with information provided by the neighbouring village. The members were disciplined according to their service contracts, losing their jobs and income from tourism for a year, in addition to being fined according to NPA regulations. After Year 2, the number of villages sharing the VDF was expanded from 9 to 14 in order to include an additional five villages that the ecotourism villages reported as being responsible for most of the continued illegal hunting in the tour area. In Year 3, there were no hunting infractions from the original nine villages, although there were two infractions by two of the newly added villages. However, these infractions were reported by the villages themselves which, according to the contract design, resulted in no reduction of their respective VDF earnings for that year. In Year 4, there was one hunting infraction overall, which was committed by one of the newly added villages. It is important to note that increases in infractions in Year 3 were not related to the behaviour of the original nine villages, but rather the result of adding new villages to the program who had not yet modified their hunting behaviour. There was a decline in infractions in the five new villages in Year 4, their second year in the program, which was similar to that observed in the original nine villages in their second year of involvement in the program.

**Fig 6:**
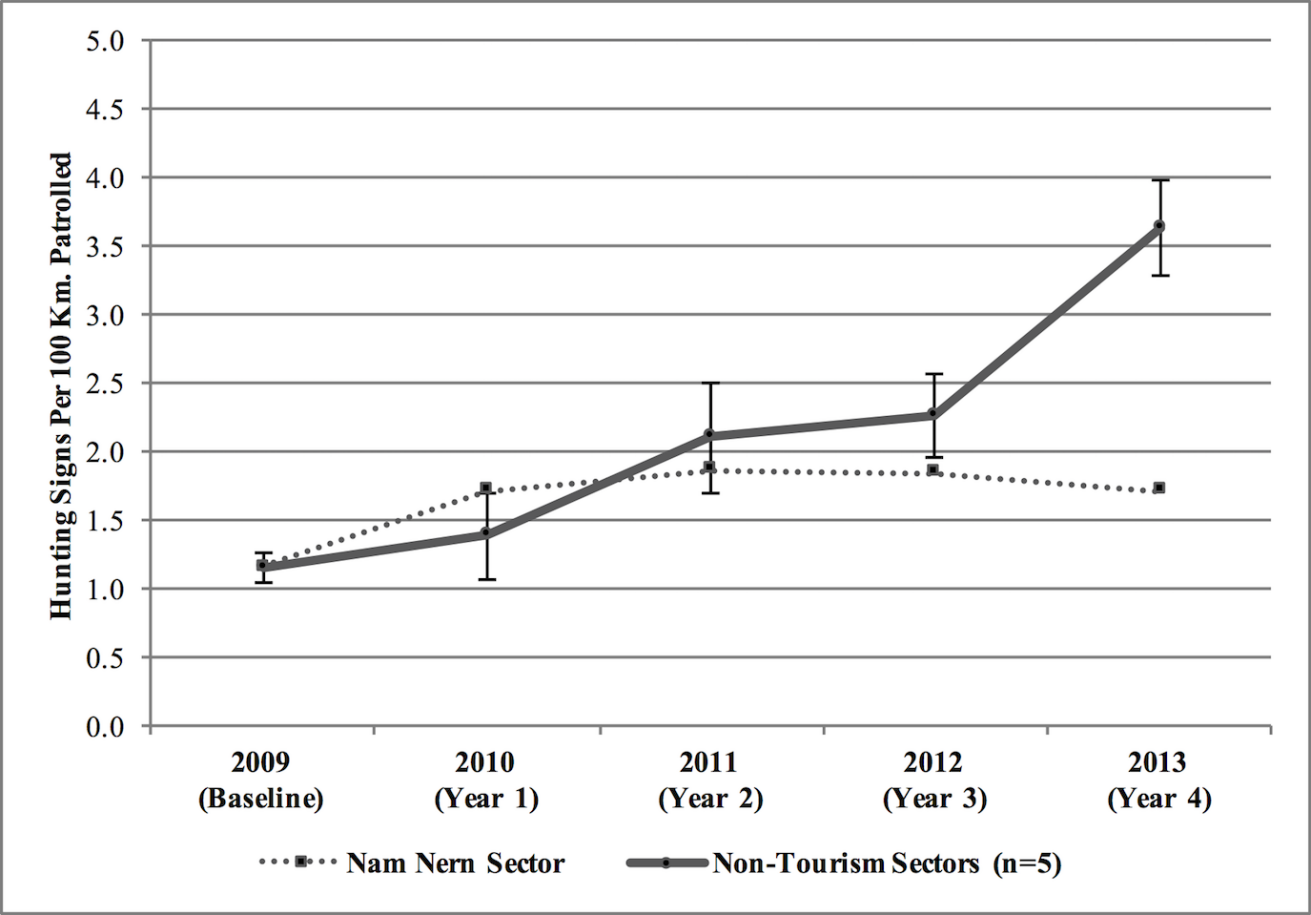
Trend in hunting catch per unit effort in the Nam Nern sector of the TPZ relative to mean hunting catch per unit effort for the five sectors without ecotourism activities in the TPZ from 2009–2013.

Law enforcement monitoring in the Nam Nern sector, where the ecotourism strategy was implemented, indicated that hunting CPU in Year 4 was no greater than in Year 1 whereas there had been a three-fold increase in mean hunting CPU in the five non-tourism sectors of the TPZ during this same period [Fig 6].

#### Status of biodiversity

**Fig 7:**
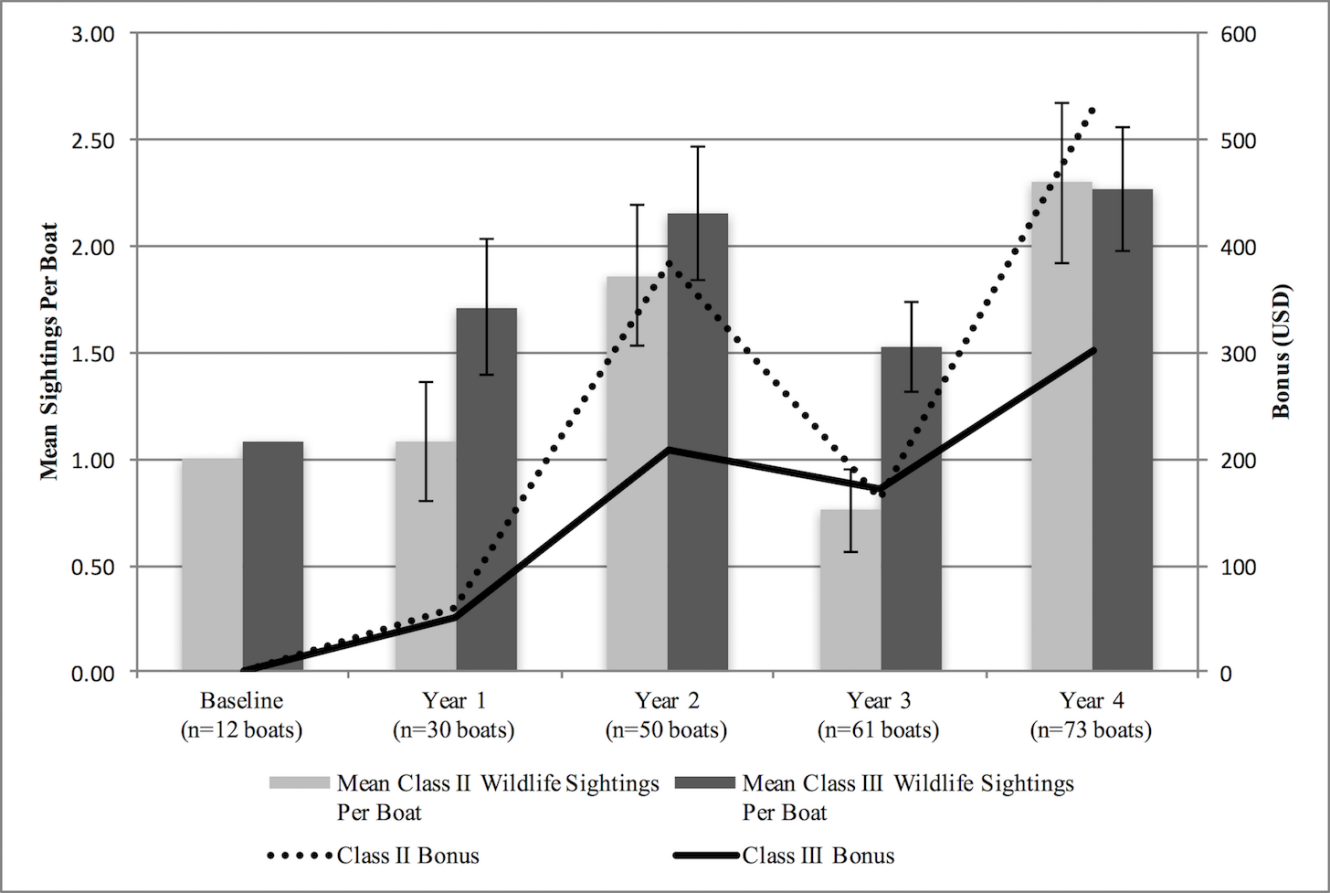
Mean number of sightings of Class 2 and Class 3 wildlife per boat and total bonus money paid for wildlife sightings to the nine villages over a four-year period (2009–2013).

A total of 214 boats participated in the Nam Nern Night Safari from Years 1–4 (2010–2013. During this period, the mean number of wildlife sightings per boat of Class II and Class III species combined (not including tiger tracks) increased by 63% from the Year 1 to Year 4 (2.80 and 4.56, respectively) [Fig 7].

From Year 1 to Year 4, there was a total of 260 sightings of Class II species (range 27–121 per year) and 356 sightings of Class III species (range 46–138 per year), which generated a total of USD1,879 in bonus money for the VDF (range USD113-831 per year; Fig 7). After Year 1, the VDF fee and prices for wildlife viewings were doubled to increase the economic incentive for wildlife protection. Although tiger tracks were seen by tourists each year, there were no tiger sightings.

## Discussion

After the first four years, the initial results of this direct payments approach suggest that the ecotourism strategy was achieving the objectives of increasing village and NPA income from ecotourism and was potentially contributing to a reduction in illegal hunting and an increase in wildlife sightings in the ecotourism area. Increases in the average number of wildlife sightings per boat and a relative decline in hunting infractions supported our hypothesis that wildlife observations would increase and illegal hunting would be reduced in conjunction with participating villages being rewarded for the wildlife sightings. Given the relatively short duration of the study and small sample sizes, these results, while promising, should be viewed with caution. In the following section, various factors that may have contributed to the observed results are discussed and recommendations for model improvement are provided.

The rise in wildlife observations during these first years was likely attributed to wildlife becoming less wary of human presence and more habituated to tourists than to an actual increase in wildlife abundance. It is known from many protected areas that increased safety from hunting in a tour area can make it easier to see animals [18]. In this case, animals may have congregated along the river without a net increase in the population (leakage of wildlife from surrounding areas to the tour area). Only over a longer period of time would this habituation be expected to become normalized and for reproduction to take effect such that increased wildlife sightings could be attributed to an increase in wildlife abundance. Wildlife habituation in the tour area may have indicated short-term success in threat reduction, but may not have equated to decreased threats in the TPZ overall. Tourist presence could potentially scare hunters away from the immediate tour area to other, less-visited areas in the sector (i.e., areas without tourists). In such a case, the likelihood of seeing wildlife would have increased in the immediate tour area and decreased elsewhere in the sector where hunters were concentrating, with no net reduction in threats—thus inflating the conservation value of ecotourism.

To determine if the lack of disturbance along the tour route was a result of an overall reduction in threats in the TPZ, hunting CPU for the entire Nam Nern sector as well as the number of infractions by villages involved in ecotourism were examined. If threats in the overall sector were decreasing while wildlife sightings were increasing, then it could be assumed that there was no leakage and that there was a net positive benefit to conservation. In this case, results indicated that wildlife sightings had increased in the ecotourism area overall while increases in hunting CPU slowed in the Nam Nern sector in comparison to other sectors of the TPZ. Therefore, assuming that villagers from the tourism area did not travel extraordinary distances to hunt in other sectors of the TPZ—a plausible assumption, described and supported by data presented in section 1.1—the data seemed to point to ecotourism having had a positive net effect on reducing hunting threats in the TPZ overall. Furthermore on the issue of leakage of hunters from the Nam Nern sector to other sectors, which would reduce CPU without reducing hunting in the protected area overall, the incentives created by the benefit sharing contract would not give hunters any added incentive to travel further to other sectors since their village would lose benefits if they were caught regardless. The demonstrated reduction in infractions by all ecotourism villages and the slower growth of hunting threats in the sector of the TPZ with ecotourism during the study period presents a positive result that lends support for the use of an ecotourism strategy with a direct payments approach.

Underpinning the hunting CPU index is an assumption that quality of enforcement effort was constant (i.e. for every kilometre patrolled the probability of finding a threat is based solely on the actual number of threats). It is important to acknowledge that detection was imperfect and hunting CPU could vary in the short-term with personnel changes of a patrol team or management decisions that affect team morale and work ethic [43]. For example, during the study period there were multiple changes in enforcement personnel of the Nam Nern sector as well as management decisions that affected enforcement teams throughout the NPA [24]. Another issue to consider is inadequate reporting. A leader of the Nam Nern sector patrol team was moved in 2013 on suspicion of misreporting and poor conduct. He was from the main tourism village and would have lost personal economic benefits for catching hunters from his own village. Although there was no disincentive for the patrol team to underreport general signs of hunting, which would affect the CPU estimates, there was the possibility that hunters were not caught and reported, especially from Son Koua.

In Year 3, wildlife sightings declined, while hunting CPU and infractions in the ecotourism area remained constant. According to verbal accounts by villagers, this drop was partially attributed to the district government’s decision to permit a company to engage in alluvial gold mining on one of the tributaries of the Nam Nern River (an issue which has been on the increase throughout the country [44]). This reportedly led to reduced fish harvests and undermined people’s faith in the government’s commitment to the ecotourism agreement, which had contributed to an increase of hunters in the tour area. The company left the area at the beginning of Year 4, which was partly a result of complaints made by the project. The reported issue highlighted a serious oversight in the model in that the economic benefits for government stakeholders from ecotourism accrued only to the NPA office (in the form of the park entrance permit) and not to the district government—which is a more powerful entity than the NPA. Thus, the district government had no direct economic incentive to uphold its responsibility to forbid mining or other conflicting land uses in the tour area, as stipulated in the ecotourism agreement,. The benefits accrued to the government were both nominally and relatively low in comparison to village benefits. Furthermore, unlike the villages’ benefits, the government’s benefits were not pegged to wildlife sightings, creating no direct incentive for the government to implement measures to increase wildlife sightings. In response to this oversight, a fee for the district government and an increase for the protected area’s fee were instituted after Year 4. These issues illustrated a general weakness with conservation strategies that are focused on community-only solutions towards managing natural resources for which the government holds authoritative rights [45] and the value of using a theory of change and monitoring results to inform regular review and adaptive management [39].

Another important issue to explore is that of protecting the rarest wildlife species, in this case tigers, with a direct payments ecotourism model. In our case, tourists did not see a tiger over the four-year period, and therefore no money was paid into the VDF for seeing tigers. Although tourists did see tiger tracks annually, which added a small amount of money to the VDF, villages did not experience the potentially powerful positive incentive of receiving the relatively large USD225 bonus for a tiger sighting. To compensate for this, the ecotourism strategy should have been adapted to create a much larger bonus for tiger track sightings. Even then, a direct payments scheme would likely leverage limited protection for a species that is too rare to be seen and so highly valued by wildlife traffickers. Although tigers declined in the NPA from an estimated 7–23 in 2006 to only two tigers detected in 2012 [31], it is important to note that one of these two tigers was recorded in the Nam Nern sector.

Even for the conservation of wildlife other than tigers, an issue that pervaded the effectiveness of the model was that the benefits from ecotourism were still quite low, which was a function of developing tourism in a remote region with low visitation but yet the only likely place to see large wildlife in the country. For some in the villages, ecotourism’s benefit was lower than the expected individual gain from the illegal harvest and trade of wildlife less the risk of being caught, as suggested by the infractions committed. Although it appears that this direct payments model did change behaviour with at least some individuals and contribute to conservation, it was not expected to act as the single strategy to protect wildlife from illegal hunting. In most protected areas where illegal hunting is a threat, an ecotourism strategy is implemented alongside a law enforcement strategy [11–14]. In response to the leopard/tiger killing in Year 2, an adjustment to the benefit-sharing contract was made (by popular vote) to increase the penalty for poaching protected species to a 100% loss of the VDF. Although this change may help, as with many other enterprise strategies for community-based conservation [37] the ecotourism program should clearly not be seen as a silver bullet, but instead should be treated as a supplementary strategy supporting other existing NPA strategies (e.g. law enforcement and outreach). That being said, the model could be further adapted to create a greater connection between wildlife sightings and ecotourism benefits so as to maximize its potential effectiveness. Only half of the VDF, representing only 6–8% of total ecotourism revenue (see Fig 4) was pegged to wildlife sightings. Adapting the strategy to pay bonuses for wildlife sightings to tourism service groups ***and*** government stakeholders may further increase the strategy’s effectiveness, or at least allow for a more robust test of the direct payments approach. In response to this issue, after Year 4 bonuses for wildlife sightings were included as part of village tourism service group wages, pegging 10–25% of wage income to wildlife sightings by paying members a bonus for wildlife sightings.

One remaining question about the model is whether or not assigning different values to wildlife according to their rarity increased the effectiveness of the strategy. We would expect that Class II sightings would have fluctuated less per annum as a result of their higher bonus rate. Instead however, Class II sightings were quite volatile over the four years, while Class III species sightings exhibited less dramatic changes. Looking closer at the data, it can be seen that the species most responsible for the sharp rise and fall in Class II numbers was Sambar deer, which is one of the principle prey species of tiger and perhaps both the most desirable and easiest of animals for villagers to hunt along the river due to its large size and propensity to regularly visit known mineral licks along the river bank. Sambar deer numbers are consequently very sensitive to changes in hunting in the tour area. Any change in hunting would likely impact Sambar numbers first and foremost. This does not disprove the fact that the higher bonuses for Sambar deer did not provide any relief from overall decline in wildlife sightings in Year 3. However, it may have been worth experimenting with an even higher bonus rate for Sambar given that it is targeted by hunters and as well as being an important tourism attraction and a large carnivore prey species.

Some would argue that it may have been more appropriate to base bonus values on species’ black market prices, as were the NPA fines [46] to more adequately reflect species’ values. However, a bonus would still realistically only represent a fraction of a species’ market value, as tourists would likely not be willing and able to pay the entire market price—especially for the rarest of species, some of which often have rates higher than the tour price itself[28]. Alternatively, the price for a sighting could be calculated as a portion of the total market price using a few key factors: the probability of seeing the species each tour multiplied by the estimated number of tours (or boats) per year, divided by the timeframe in which stakeholders would expect to be rewarded for anti-poaching, i.e. the opportunity cost of not hunting. For example, a Sambar deer bonus was about 1% of what a hunted Sambar deer was worth on the local black market, requiring that tourists see the deer and pay the bonus more than 100 times before stakeholders could recover the opportunity cost of not hunting. The rate could, therefore, be regarded as too low if one believed that the opportunity cost should be met within one tourism season. Basing bonuses on black market rates would increase the complexity of the payments system, however, as each species would require its own rate—a foreseeable drawback for negotiating community contracts. It would be useful for future studies to gather data on villagers’ perceptions of what is a reasonable bonus before deciding on prices.

Finally, we would like to address the issue of how ecotourism can provide some basic information necessary to judge its effectiveness as a conservation strategy. This model presented a simple wildlife monitoring system that used local guides and tourists as data collectors, which proved useful and relatively easy to implement. By repeatedly following the same route during the same general hours, the tour was essentially a transect. Using experienced local guides, who were former hunters, to record numbers of wildlife observations during tours along with tourists verifying the sightings, to reduce over-estimation or cheating, was a no-cost solution that provided protected area managers with some of the basic information necessary to assess the biodiversity benefits of the ecotourism strategy. This system could easily be replicated elsewhere and should be where projects have insufficient funding or technical expertise to conduct regular independent wildlife surveys—which is especially true for private sector projects with no outside support. Although the tour represented just one location in the NPA and, as a result, did not represent the status of wildlife in the entire area, it would serve as a good indicator of wildlife status in that particular area, especially if done over a long enough time period to adjust for habituation. For projects that also have a law enforcement strategy in place, threat monitoring data can contribute to a more robust evaluation of the effectiveness of ecotourism than wildlife sightings alone, especially if monitoring is done over an area that is greater than the tour route, making it possible to assess overall net impact. Hunting CPU may, however, be too technical for rural communities to understand when negotiating community-based direct payment agreements, in which case infractions would be the preferred threat indicator.

## Conclusion

The assumptions of this ecotourism strategy were that increased income from payments for wildlife sightings would reduce threats of illegal hunting and trade and ultimately increase wildlife sightings as an indicator of wildlife abundance. The preliminary results showed positive support for these assumptions although it is important to maintain caution when drawing conclusions due to a number of potential factors other than ecotourism that may have contributed to the observed outcomes. The results indicated a negative correlation between ecotourism income and hunting infractions and that threats to wildlife slowed in the ecotourism sector of the protected area relative to non-tourism sectors, although trends in wildlife sightings continued to fluctuate. The results illustrate how an ecotourism strategy using direct payments for wildlife sightings, along with a simple wildlife monitoring system can augment an enforcement strategy to reduce the threat of illegal hunting and trade. The preliminary results of this direct payments model for ecosystem services provide lessons for adapting and testing the model elsewhere, including the need to provide significant benefits to non-community stakeholders who have rights to the resource; to balance the relative value of incentives with other tourism income; and to consider incentive rates that can create significant value for the rarest species based on perceived stakeholder opportunity costs.

## Supporting information

S1 File Data for creating figures. (XLS)

S2 File Infractions data. (XLSX)
